# Evolutionary Emergence of Drug Resistance in *Candida* Opportunistic Pathogens

**DOI:** 10.3390/genes9090461

**Published:** 2018-09-19

**Authors:** Ewa Ksiezopolska, Toni Gabaldón

**Affiliations:** 1 Bioinformatics and Genomics Programme, Centre for Genomic Regulation (CRG), The Barcelona Institute of Science and Technology (BIST), 08003 Barcelona, Spain; ewa.ksiezopolska@crg.eu; 2 Universitat Pompeu Fabra (UPF), 08003 Barcelona, Spain; 3 Institució Catalana de Recerca i Estudis Avançats (ICREA), 08010 Barcelona, Spain

**Keywords:** *Candida*, antifungal drugs, drug resistance, evolution

## Abstract

Fungal infections, such as candidiasis caused by *Candida*, pose a problem of growing medical concern. In developed countries, the incidence of *Candida* infections is increasing due to the higher survival of susceptible populations, such as immunocompromised patients or the elderly. Existing treatment options are limited to few antifungal drug families with efficacies that vary depending on the infecting species. In this context, the emergence and spread of resistant *Candida* isolates are being increasingly reported. Understanding how resistance can evolve within naturally susceptible species is key to developing novel, more effective treatment strategies. However, in contrast to the situation of antibiotic resistance in bacteria, few studies have focused on the evolutionary mechanisms leading to drug resistance in fungal species. In this review, we will survey and discuss current knowledge on the genetic bases of resistance to antifungal drugs in *Candida* opportunistic pathogens. We will do so from an evolutionary genomics perspective, focusing on the possible evolutionary paths that may lead to the emergence and selection of the resistant phenotype. Finally, we will discuss the potential of future studies enabled by current developments in sequencing technologies, in vitro evolution approaches, and the analysis of serial clinical isolates.

## 1. Introduction

From the estimated 1.5 million fungal species, around 300 have been reported to present virulence towards humans, even if sporadically [1]. Fungal pathogens can cause life threatening invasive infections (e.g., fungaemia, meningitis), chronic conditions (e.g., pulmonary aspergillosis, asthma), and recurrent superficial infections (e.g., oral and vaginal candidiasis). Globally, fungi can affect millions of people every year, and the overall death toll has been estimated to be around 1,350,000 deaths per year [2]. Species belonging to the genera *Candida*, *Aspergillus*, and *Cryptococcus* are the most prevalent cause of invasive infections, with *Candida* being responsible for the most common invasive fungal disease in developed countries—candidiasis [3]. Population-based studies have estimated the incidence rate of candidiasis to be two to 14 cases per 100,000 inhabitants, and candidemia (*Candida* bloodstream infection) affects more than 250,000 persons worldwide every year, leading to more than 50,000 deaths [4]. In addition, candidemia brings a substantial economic burden, involving, on average, three to 13 days of hospitalization in the US, with total associated costs ranging from $6000 to $29,000 [5]. A large study of more than 1800 clinical fungal isolates from 31 countries found that 82% of the fungal infections in 2013 were caused by *Candida* [6]. Currently, the effective treatment of candidiasis is limited by two major factors, namely the difficulty of fast and accurate diagnostics of the invasive agent, and the limited number of therapeutic options. 

Candidiasis is usually diagnosed late. Firstly, fungal infections are generally considered only after antibiotic treatments fail to reduce fever. Secondly, standard diagnostic approaches require blood cultures, which are slow and can have a low sensitivity. For instance, some studies reported sensitivities as low as 17% [7] or 45% [8]. Furthermore, although the four most common *Candida* (*C. albicans*, *C. glabrata*, *C. parapsilosis*, and *C. tropicalis*) can account for more than 80% of the cases, there is a long list with over 30 *Candida* that have been identified as candidemia agents [9], and the list keeps expanding. Added to the difficulty of a fast and accurate diagnosis, doctors face severe limitations with regards to treatment options. Currently, there are only four major classes of antifungals in clinical use: azoles, polyenes, echinocandins, and pyrimidine analogs [10]. This situation alarmingly decreases the chances of a successful treatment and increases the possibilities of a fatal outcome if the infecting pathogen is resistant to one or multiple drugs. Limitations in diagnostic methods further enhance the problems of a few therapeutic options, as different species may show diverse resistance profiles. Thus, diagnostics of the infecting agent, along with susceptibility tests, should be used to inform the choice of therapy (discussed below). Over the last years, the intensive use of some antifungal drugs, such as azoles, has promoted a shift in the epidemiology of candidiasis, in which the incidence of *C. albicans* has decreased in favor of other species that are naturally less susceptible to this drug, such as *C. glabrata*. 

To the problem of the intrinsic variation of drug susceptibility among different *Candida,* we need to add the emerging issue of acquired resistance, which refers to the ability of yeasts to evolutionarily develop mechanisms that lower their susceptibility towards a given drug [11]. This process generally involves mutations ranging from chromosomal re-arrangements to point mutations. These mutations can affect drug resistance in different ways, ranging from directly interfering with the binding of the drug to its target to inducing gene expression changes that promote physiological states that reduce drug susceptibility. In this regard, an enhanced capacity to form biofilms can result in the acquisition of resistance, as these structures promote yeast survival upon exposure to the drug [12,13].

The emergence of resistant strains, including those becoming resistant to multiple drugs, has been increasingly reported in recent years [14,15,16]. In addition, it has been demonstrated that such resistant phenotypes can develop over the course of an infection, and in response to treatment, which adds yet another threat to patients [17].

Despite the clinical and economic relevance of drug resistance in the context of yeast infections, this subject remains poorly studied, at least in comparison with the similar issue of antibiotic resistance in bacterial pathogens. Although parallels can be established, the evolutionary mechanisms underlying the emergence of resistance in fungi and bacteria are markedly different. While drug resistance in bacteria generally involves the transference, between strains or species, of genetically mobile elements such as genomic islands [18], in fungi, resistance commonly appears via genetic alterations within a lineage. Still, we are far from having a broad understanding of how resistance towards antifungal drugs emerges in the context of infection or commensalism in yeast pathogens. Fortunately, recent developments in sequencing technologies are enabling us to catalog and trace the origins of mutations conferring resistance to antifungal drugs in different species. In this review, we aim to summarize our current knowledge on how drug resistance is genetically determined in *Candida* opportunistic pathogens, and how it can be acquired in the course of evolution. In doing so, we will focus on how the advent of genomics technologies is allowing us to study these processes on unprecedented levels of scale and resolution, and how possible future studies could help us further our understanding of the evolutionary emergence of drug resistance in yeasts.

## 2. Major Antifungal Drugs and Their Mechanisms of Actions

**Figure 1:**
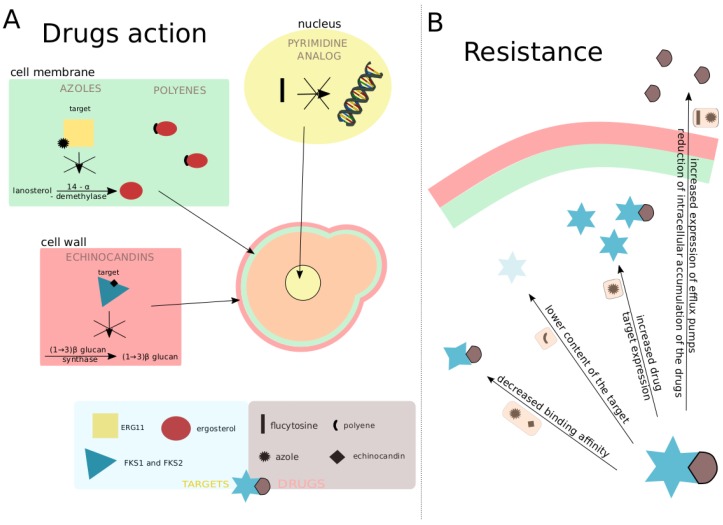
Antifungal drug actions and resistance mechanisms in *Candida*. (**A**)—action mechanisms of azoles, polyenes, echinocandins, and the pyrimidine analog in different parts of the cell. Colored shapes indicate target enzymes or molecules, with the name of the coding gene or the molecule, respectively, indicated in the light blue box at the bottom. Black shapes indicate different drug classes and a pyrimidine analog, flucytosine, with their correspondence indicated in the light brown box at the bottom. Mechanisms of actions are schematically indicated (see text) with colors and arrows indicating the main cellular location of the effect of the drug. (**B**)—most common resistance mechanisms caused by mutations. Targets are generically represented by blue stars and drugs by a brown shape. Different mechanisms causing resistance are indicated by arrows with light orange boxes indicating types of drugs for which this mechanism has been observed. Drug shapes are as in A.

The development of new antifungal drugs is challenging, as fungi are eukaryotic organisms that share many basic cellular processes with us. This evolutionary relatedness makes the finding of specific targets difficult and increases the likelihood of undesired secondary effects. Existing antimycotic drugs target processes that are highly divergent between fungi and the human host, such as the ergosterol synthesis pathway. Here, we will briefly summarize the main mechanisms of action of the major antifungal drug classes (Table 1, Figure 1A). 

Azoles are heterocyclic compounds containing at least one nitrogen atom as part of the ring. Common azoles used as antimycotic agents include the triazoles: fluconazole, voriconazole, and posaconazole. These drugs act by targeting the cytochrome P450 enzyme-lanosterol 14α-demethylase, that converts lanosterol to ergosterol. In yeast, this enzyme is encoded by the *ERG11* gene. Similar to cholesterol in animals, ergosterol is the main membrane sterol in most fungal species, holding an important role in controlling membrane fluidity [19]. As a result of the action of azoles, the *Candida* cell membrane is depleted of ergosterol and accumulates other toxic 14α-methylated sterols. Subsequently, this causes the decrease in membrane fluidity and, in most of the cases, inhibits cell growth [20]. Fluconazole is the azole drug most widely used for the treatment of *Candida* infections. Its utility is attributed to its high bioavailability, high water solubility, and low affinity to plasma proteins [21]. Unfortunately, the fungistatic character of fluconazole and its extended, perhaps excessive, use is inevitably leading to an increasing selection in favor of resistant yeast isolates. 

Echinocandins are amphiphilic lipopeptides, and products of cyclopentamine. They can be formed during the fermentation of some fungi such as *Zalerion arboricola* or *Aspergillus nidulans* var. *echinulatus*, but nowadays, they are produced semi-synthetically for clinical use. The most common representatives of this class of drugs are: caspofungin, micafungin, and anidulafungin. Echinocandins inhibit the biosynthesis of an essential component of the fungal cell wall, the 1,3-ß-glucan. In *Candida*, they target two subunits of the 1,3-ß-glucan synthase, encoded by the *FKS1* and *FKS2* genes [22], and eventually cause cell lysis. The fungicidal character against most *Candida*, their target not being present in mammalian cells, the lack of clinically significant drug-drug interactions, and the absence of adverse effects make this antifungal drug class considerably attractive for the treatment of fungal infections. Echinocandins were approved for medical use in 2002 and they are applied as a first line antifungal drug along with fluconazole. Due to their safety profile, better outcomes, and the emergence of azole-resistant species, echinocandins are currently the preferred agents for most episodes of candidemia and invasive candidiasis, with the exception of those affecting the central nervous system, the eye, and the urinary tract [23]. 

Polyenes are poly-unsaturated organic compounds that contain at least three alternating double and single carbon–carbon bonds. Their antimycotic action is mediated by direct binding to and removal of ergosterol present in the fungal cell membrane. This results in the loss of membrane permeability, subsequent membrane leakage, and eventually cell death [24]. In the 1950s, the polyene amphotericin B deoxycholate was the first approved successful antifungal drug [25]. Nowadays, amphotericin B continues to be broadly used despite its high toxicity, which results from structural similarities between ergosterol and human cholesterol. Due to this toxicity, the use of amphotericin B in high concentrations may be harmful and cause damage to human tissues, such as the kidneys [26]. 

Pyrimidine analogs are nucleosides that mimic the structure of natural pyrimidines. The only pyrimidine analog with antimycotic properties currently in use for human treatment is flucytosine, which has the potential to convert into 5-fluorouracyl and further to 5-fluorodeoxyuridine inside the fungal cell [27]. Subsequently, 5-fluorodeoxyuridine interferes with DNA, RNA, and protein synthesis. The transformation of flucytosine into 5-fluorouracyl is catalyzed by the action of the fungal enzyme cytosine deaminase (encoded by the yeast gene *FCY1*), which is not present in humans. Although the most effective and safest antimycotic in the health system [28], it is not used in monotherapy due to the rapid development of resistance towards this drug [24,29].

## 3. Natural Susceptibility to Antifungals among *Candida*

Out of the 30 different species of *Candida* able to infect humans, *C. albicans*, *C. glabrata*, *C. parapsilosis*, and *C. tropicalis,* generally in this order, account for up to 80% of candidiasis cases. Although infections with *C. albicans* are still the most common, epidemiology is shifting towards *non-albicans Candida*, wherein the specific relative incidences are being time- and space-dependent [30] (for detailed geographical variation see [31]). When highlighted on a phylogenetic tree, *Candida* opportunistic pathogens belong to distinct lineages, which are interspersed with non-pathogenic relatives [9]. This implies that the ability to infect humans emerged several independent times during evolution. As a consequence, different *Candida* may use different mechanisms for evasion of the host immune system and exhibit different virulence-related phenotypes [32]. Accordingly, various *Candida* present distinct susceptibility profiles towards antifungal drugs and different trajectories to acquire resistance when exposed to antifungals. Here, we will briefly survey known antifungal susceptibility characteristics of the main *Candida* pathogens.

How microorganisms respond to a drug is assessed experimentally by means of susceptibility tests. Levels of susceptibilities are indicated by the minimum inhibitory concentration (MIC), which is defined as the lowest concentration of the tested compound at which 50% (MIC 50), 90% (MIC 90), or complete growth inhibition of the microorganism is observed. Susceptibility tests are commonly used in epidemiological studies, in studies comparing in vitro activities of existing and new antimycotic drugs, in guiding therapy strategy, and in monitoring the emergence of resistance. 

Epidemiological studies, performed on globally sampled clinical isolates, reveal differential susceptibility patterns among *Candida*. They indicate how frequently isolates of a species are resistant to different drugs, which reflects intrinsic characteristics of the species (Table 2). For instance, *C. glabrata* and *C. krusei* have a naturally low susceptibility to azoles, while *C. parapsilosis* strains tend to have a lower susceptibility to echinocandins [33]. There is a growing number of rarely occurring *Candida* being reported to have lower susceptibilities to one or several drugs. Species naturally more tolerant to azoles include the above mentioned *C. glabrata* and *C. krusei*, as well as a long list of less common species such as *C. ciferrii*, *C. guilliermondii*, *C. inconspicua*, *C. humicola*, *C. lambica*, *C. lipolytica*, *C. norvegensis*, *C. palmioleophila*, *C. rugosa*, and *C. valida*. Among the species more tolerant to echinocandins, besides *C. parapsilosis*, we can find *C. orthopsilosis*, *C. metapsilosis*, *C. guillerimondii*, *C. lipolytica*, and *C. fermentati* [33,34,35]. Finally, *C. lusitaniae*, *C. guilliermondii*, *C. glabrata*, and *C. krusei* have a generally lower susceptibility to polyenes [36,37]. Importantly, an intrinsic multidrug resistant *Candida auris* has been recently reported as an emerging cause of healthcare-associated infections worldwide in at least a dozen countries on four continents during 2009–2015 [38]. Infections caused by this species can have high mortality rates ranging from 30–60% [39]. Very often, strains of this emerging species are resistant to the three major drug classes: polyenes, azoles, and echinocandins. Indeed, up to 96% of *C. auris* may exhibit resistance to fluconazole, an exceptionally high value compared to 0.5–2% for *C. albicans*, 4–9% for *C. tropicalis*, 2–6% for *C. parapsilosis*, and 11–13% for *C. glabrata* [16,40,41]. For these reasons, *C. auris* has been highlighted by the American and European centers for disease control (CDC and ECDC) as a cause of major concern. 

## 4. Epidemiological Studies Report Increasing Levels of Resistance

Worryingly, the picture of resistance levels across *Candida* isolates is not a static one. Rather, epidemiological studies are showing a steady rise in the amount of reported resistant isolates, even among naturally susceptible species. For example, an increase in fluconazole resistance in naturally susceptible species such as *C. parapsilosis*, *C. guilliermondii*, *C. lusitaniae*, *C. sake*, and *C. pelliculosa* was observed in a population-based surveillance programme comprising more than 250,000 *Candida* strains isolated between 1997 and 2007 [43]. Often, for naturally susceptible species, both the relative amount of resistant strains and the overall MIC levels in clinical isolates increase after the continuous use of a given antifungal drug [44]. Furthermore, the acquisition of resistance towards one drug in species that are intrinsically resistant to another one is not uncommon and leads to dangerous multidrug resistance (MDR). An example of this would be the acquisition of resistance to echinocandins by species like *C. glabrata* or *C. krusei*, which already exhibit a lower natural susceptibility towards azoles. The increased use of antifungals during the last 15 years correlates with an alarming development of MDR, especially in *C. glabrata* [45]. For example, a large study assessing more than 1300 isolates from 80 USA hospitals indicated that 32.9% of the *C. glabrata* isolates classified as non-susceptible to echinocandins were also resistant to fluconazole, and that, overall, 1.7% of the strains presented MDR [46]. Similarly, the CDC/SENTRY antimicrobial surveillance program reported a rise from 0 to 11% in the fraction of fluconazole-resistant strains that were also less susceptible to echinocandins between the studies performed in 2001–2004 and 2006–2010 [47]. Other studies on *C. glabrata* report that 14% of fluconazole-resistant strains exhibit resistance to at least one echinocandin and a total of 3.5% of MDR cases were noted in Duke University hospital [48], 7% of MDR at MD Anderson Cancer Center [45], and a resistance to azoles in 36% echinocandin-resistant strains was indicated in a five-year surveillance study in the USA [49]. Importantly, instances of cross-resistance towards amphortericin B and azoles or echinocandinds in *Candida* have also been reported [50,51,52,53].

Other studies have shown that while the fraction of *C. glabrata* infecting strains resistant to caspofungin in the United States is significant (10%) [45], in Europe, it is much lower, with 0% reported in studies performed in Italy and Spain [54]; 2.1% in Lombardy, Italy [55]; and 2% in Turkey [56]. These differences in distribution of the resistant *Candida* may result from regional differences in either species or strain distributions or in antifungal use and prophylaxis protocols. MDR in species with no intrinsic tolerance to drugs is rare, probably because it requires multiple steps, each associated with a fitness cost. However, MDR is not restricted to *C. glabrata* or *C. auris*. Other examples include *C. kefyr* [57], *C. lusitaniae* [58], and *C. albicans* [59,60,61]. Hence, the threat is real, and instances of increasing occurrence, natural resistance, and ease in acquisition of resistance should raise much more awareness. Azoles and echinocandins are the two most used antifungal drugs in hospitals, and the emergence of combined resistance to both of them severely hampers our ability to treat fungal infections. 

## 5. Mutations Leading to Secondary Acquisition of Resistance

High genomic plasticity is one of the characteristics of *Candida* yeasts that enables their fast adaptation to varying environments [62,63,64]. Upon exposure to drugs, the yeast cell population is subjected to a strong selection towards the subset of cells that can better adapt to the stressing conditions [65]. Eventually, this selection pressure can lead to the increase in frequencies of mutant alleles that confer enhanced resistance to the administered drug, resulting in a population not responding to the treatment anymore. This can occur during long hospitalization periods and prolonged treatments [66]. Besides the overall use and exposure to antifungals (often used in prophylactic measures), other factors that promote the acquisition of resistance and a treatment failure include the use of sub-therapeutic concentrations, drug sequestration in the biofilm matrix, and poor control of infections [67,68]. Mechanisms of acquired resistance mostly fall into two classes (Figure 1B): (i) mutations leading to increased expression of the target or the alteration of its binding affinity towards the drug; and (ii) mutations leading to reduced intracellular accumulation of the drug by means of increasing the activity or expression of drug efflux pumps or, conversely, reducing the import of the drug [69]. Below, we survey the current knowledge on known mechanisms of resistance towards the main classes of antimycotics (Table 3, Figure 1B).

Resistance towards azoles can involve various mechanisms, namely: (i) changes in the biosynthesis of sterols, resulting in their substitution for ergosterol; (ii) overexpression of the target enzyme, leading to sufficient levels of activity in the presence of the antifungal drug; (iii) overexpression of drug efflux pumps that diminish the intracellular concentration of the drug; and (iv) changes in the target gene sequence, leading to the reduction in the binding affinity of the protein to the drug [69,70]. Acquired resistance to this group of antimycotics seems to be a result of mutations selected by the pressure exerted by the drug [13]. The adaptation is said to appear gradually during continuous contact with the antifungal [13]. In *C. albicans*, acquisition of resistance is often related to point mutations in the *ERG11* gene, encoding the enzyme targeted by azoles [71,72]. Out of 140 different point mutations described for this gene, 21 have been directly associated with fluconazole resistance [73]. Additionally, inactivation of the protein encoded by the *ERG3* gene has also been found to confer azole resistance [59]. Furthermore, *ERG3* mutations result in the reduction of ergosterol and accumulation of other sterols, often leading to cross-resistance to polyenes [74]. Other factors contributing to decreased susceptibility to azoles in *C. albicans* involve the increased expression of *ERG11* due to activating mutations in the gene encoding its zinc-finger transcriptional regulator *UPC2* [75]; overexpression of the drug efflux pumps, including multidrug resistance gene *MDR1* (controlled by the transcription factor *MRR1*) [76]; or Candida drug resistance 1 and Candida resistance 2 *(CDR1/CDR2)* genes [77]. Importantly, the deletion of either of the *CDR1/2* genes leads to the loss of the resistance phenotype [78], and the upregulation of these pumps can be attributed to at least 17 different mutations in their transcriptional regulator *TAC1* [79,80]. Finally, gross genomic changes such as aneuploidy or the loss of heterozygosity have also been associated with increased azole resistance in *C. albicans*. For instance, aneuploidy in chromosome 5, containing *ERG11*, its transcriptional regulator *UPC2*, and the efflux pump regulator *TAC1*, results in altered susceptibilities [81], as is also the case for the loss of heterozygosity in regions encoding *ERG11*, *TAC1*, or *MRR1* [81,82]. Another recent study added elevated copy numbers of chromosomes 3 and 6 to the list of genome rearrangements associated with fluconazole resistance in *C. albicans* [83].

Resistance to azoles in *C. parapsilosis* has been attributed to mutations in the transcription factor gene *MRR1* [84] and *ERG11* (Y132F, either alone or in combination with an R398I) [85,86,87], and overexpression of *CDR1*, *MDR1*, and *ERG11* [85,86,88]. However, alternative or additional mechanisms for azole resistance may await discovery in *C. parapsilosis* [86]. For *C. tropicalis*, point mutation (again Y132F) [89], overexpression [90], and deletion mutations in *ERG11* [52] have been described as causes for azole resistance. In addition, in vitro induced resistance unveiled the presence of increased expression of multidrug transporter genes of two different families, the ABC transporters and the major facilitators, *CDR1* and *MDR1*, respectively. Yet, there is no conclusive proof that these mechanisms are acting in clinical isolates [91]. The main mechanism of resistance to azoles in *C. krusei* appears to be the reduced susceptibility of 14α-demethylase to fluconazole [92]. However, overexpression of *ERG11* [93] and the ABC transporter, *ABC1*, has also been related to fluconazole resistance in this species [94]. In addition, reduced susceptibility to other types of azoles has also been linked to point mutations in *ERG11* [95]. For *C. auris*, little is known on the precise contribution of *ERG11* mutations to fluconazole resistance. Nevertheless, some geographically distinct clades with reduced sensitivity seem to carry mutations in this gene (e.g., Y132F, K143R, and F126T), which has been implicated in reduced azole susceptibility in other species [16]. So far, there is no information on the altered expression of efflux pumps being connected with resistance in this microorganism.

In contrast to other species, azole resistance in *C. glabrata* is generally not associated with alteration in *ERG11* [33,63,96,97], but rather with mutations in the *PDR1* transcription factor, which cause the differential expression of downstream targets [97]. *PDR1* belongs to a pleiotropic-drug resistance (PDR) network of regulators responsible for the transcriptional upregulation of genes encoding drug efflux pumps, such as the *CDR1*, *CDR2*, and *SNQ2* [97,98]. Alterations in this transcription factor have been described as the main mechanism for the enhancement of azole resistance in *C. glabrata*, with efflux pumps often induced during azole therapy [99]. Another possible resistance mechanism in *C. glabrata* may involve the major facilitator superfamily (MFS) transporter, *TPO3*, as its depletion results in increased sensitivity to fluconazole and clotrimazole [100]. Alternative mechanisms for azole resistance in *C. glabratas* involve ‘petite mutants’, which are characterized by a lack of mitochondrial DNA and mitochondrial dysfunction, and which also show upregulation of ABC transporter genes, improved fitness, and increased resistance towards azoles [101]. Furthermore, mutations in 27 genes involved in transport (*PDR5* and *PDR16*), retrogade signaling (*RT2*), RNA polymerase II transcription, calcium homeostasis, ribosomal biogenesis, mitochondrial function, and cell wall signaling have been suggested to confer fluconazole resistance in *C. glabrata* [102]. Another study included calcium signaling as essential for the survival of azole treatment and its absence has the potential to change the character of fluconazole from fungistatic to fungicidal [103]. However, there may still be alternative routes to the acquisition of resistance in *C. glabrata*, as there have been at least 78 genes suggested to be implicated in *C. glabrata* resistance to fluconazole and voriconazole [104].

Acquisition of resistance towards echinocandins is not as common as towards azoles, yet it is far from rare and is significantly linked with prior exposure to the drugs [46]. In *C. glabrata*, this phenomenon has increased from 2–3% to more than 13% in a 10-year period [48] and can be present in up to one-third of isolates in the US [49]. *Candida* can evade the activity of echinocandins by mutations in particular regions (called hotspots) in the *FKS1* gene and in the case of *C. glabrata*, as well in *FKS2* (Table 4) [34]. Overall mutations in the target genes result in the reduction of the binding affinity of the antifungal drug [105]. Notably, many of these resistance-causing sequence variations are constitutive in species showing a higher intrinsic tolerance towards echinocandins (e.g., *C. parapsilosis*, *C. orthopsilosis*, *C. metapsilosis*, *C. guillerimondii*, and *C. lipolytica*) [34,35]. It has been suggested that these *FKS* polymorphisms reduce the affinity to echinocandins of the glucan synthase by two to three orders of magnitude compared to the wild-type enzyme [106,107]. What is more, the degree of susceptibility towards echinocandins depends on the position and specificity of the mutation [34]. For example, in *C. albicans*, amino acid substitutions S641P and S645Y in FKS1 and in *C. glabrata* S629P in FKS1, S663P, and F659S in FKS2 are associated with reduced activity of the drug and much higher MICs, whereas F559Y in FKS2 in *C. glabrata* reduces susceptibility to a lesser degree [42,107,108]. Additionally, in *C. glabrata*, the expression of the *FKS2* gene has been shown to be calcineurin-dependent, and the resistance phenotype can be reversed upon the application of calcineurin inhibitors such as FK506 [109]. Altered susceptibility to echinocandins is also connected with stress responses that result in paradoxical growth of the microorganism at high concentrations of the drugs and elevated cell wall chitin content [110,111].

Resistance to amphotericin B in *Candida* is still rare. When it occurs, it is generally connected with a decrease in the levels of ergosterol in the cell membrane. Lower abundance of the enzyme has been observed in polyene-resistant species, which has been attributed to mutations in *ERG2* [61,112], *ERG3* [51], *ERG5* [60], *ERG6* [113], and *ERG11* [60] genes, which encode enzymes involved in ergosterol synthesis. Decreased susceptibility towards flucytosine has been associated with point mutations in *FCY1*, *FCY2*, and *FUR1* genes and the deletion of *FPS1* and *FPS2* genes [36,114,115,116,117]. Changes in *FCY2* interfere with the drug uptake and alterations in *FCY1* and *FUR1* inactivate enzymes involved in the pyrimidine pathway, while the absence of *FPS1* and *FPS2* reduced the accumulation of the drug in the cell. Such resistance mechanisms have been observed in *C. albicans* [114], *C. lusitaniae* [115], and *C. glabrata* [116,117].

Despite the many described mutations conferring resistance to azoles or echinocandins in *Candida*, the list of possible mutations conferring resistance is probably not exhausted. Several observations suggest that unknown mechanisms remain to be discovered. For example, overexpression of the azole target gene *ERG11* has not always been associated with point mutations in its *UPC2* regulator [86,90,118], suggesting that other regulators may play a role. We also have little knowledge on the ability of the cell to uptake and transport the drugs to their targets, and so far undiscovered mutations might modulate these processes. Finally, resistant strains with no known resistance-conferring mutations in target genes have also been reported [6,69], implying the presence of yet undiscovered mechanisms. Importantly, it is not unreasonable to think that resistance might involve more than one single mechanism. Moreover, a gradient of resistance levels can exist, with some mutations conferring greater phenotypic effects than others [119]. Finally, mutations can also have synergistic or antagonistic effects with respect to the resistant phenotype. In this regard, epistatic effects between different mutations and possible synergistic effects have not been explored.

Acquired resistance limits the usefulness of species identification to define the therapeutic strategy and brings in the need to additionally perform susceptibility tests to monitor the resistance profile of the infecting strains. However, this is problematic, expensive, and time-consuming, as it requires isolation and culturing of strains before the test can be performed. Furthermore, highly standardized tests like EUCAST (European Committee on Antimicrobial Susceptibility Testing) or CSLI (Clinical and Laboratory Standards Institute) are not universally applicable. For example, these methods are not recommended for testing the susceptibility towards caspofungin (an echinocandin), given a lack of reproducibility across laboratories or even drug batches [120] and the paradoxical growth of *Candida* at concentrations above MIC [121]. In such cases, molecular methods to directly test for the presence of resistance-conferring mutations are an attractive alternative to direct susceptibility testing and, in some cases, they may even present an advantage. For instance, it has been observed that the detection of mutations in *FKS* genes has a greater predictive power than susceptibility tests regarding the risk of echinocandin therapy failure among patients infected by *C. glabrata* [122].

## 6. Evolutionary Paths for the Emergence of Resistance

**Figure 2:**
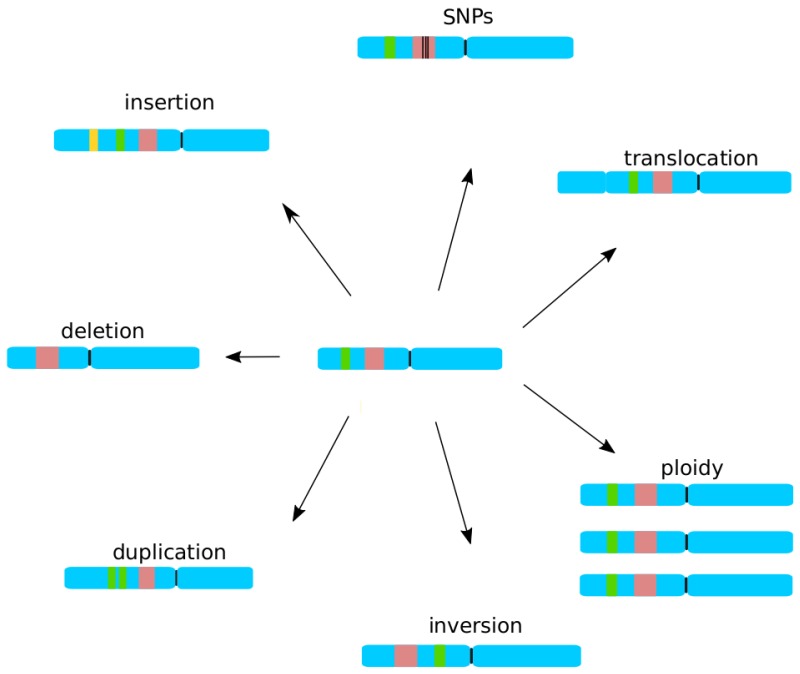
Possible genomic changes in the evolution of yeast genomes. The blue shape represents a chromosome with two arms separated by a centromere (black-line); red, green, and yellow strips represent genomic regions. The variation may be a result of single nucleotide polymorphisms (SNPs), chromosomal rearrangement (translocation or ploidies), gene-insertion, deletion, duplication, or inversion.

In contrast to the acquisition of antibiotic resistance in bacteria, the evolutionary processes by which yeasts can acquire resistance to antifungal drugs are only barely known. Cataloging mutations that can confer a resistant phenotype (see above) is only a first step towards understanding the mechanisms leading to the emergence of resistance. Processes that drive genome evolution include single-point mutations; gene duplications, deletions, inversions, and insertions; chromosomal rearrangements; aneuploidies; the loss of heterozygosity; and finally, horizontal gene transfer and/or hybridization (Figure 2). We know very little about mutation rates or frequencies of such evolutionary events in pathogenic *Candida*. Moreover, such mutations appear in the context of evolving populations, and factors such as the size of the population or the possibility of exchanging genetic material through mating and recombination, can influence the pace at which an infecting population can adapt to the drug. In addition, how a drug actually affects the pathogen may constrain the ways in which the yeast can adapt to it. For instance, fungistatic drugs that stop the growth but do not kill the pathogen open a window of opportunity for mutations to appear. Another issue contributing to the emergence of resistance involves the dosage regime. In vivo studies in mice have indicated that more frequent applications of low dosages of fluconazole, compared to less periodic and higher dosages, lead to less frequent outgrowth of resistant *C. albicans* strains [123]. Finally, various evolutionary outcomes might be driven by different selection strategies that influence the way in which the relative frequencies of drug-resistant genotypes increase within a population.

It has been suggested that some mutations or genomic re-arrangements may generally precede the appearance of point mutations, conferring a more efficient and stable resistance. Such stepwise models try to explain how resistance can appear rapidly in infecting populations that are supposedly kept at low densities by the antifungal treatment. In this regard, large genomic re-arrangements such as aneuploidies are good candidates because they result in the concerted over- or under-expression (depending on whether there is a gain or loss of chromosomes) of several genes, they are well tolerated by the cells, and they are rather common, particularly under stress conditions [124,125,126]. For example, azole resistance in *C. albicans* has been associated with a specific segmental aneuploidy comprising the two left arms of chromosome 5 flanked by a single centromere, an isochromosome 5L [i(5L)] [82]. This region carries the *ERG11* and *TAC1* genes involved, respectively, in ergosterol synthesis and drug efflux [127]. Interestingly, the acquisition of aneuploidies during in vitro evolution experiments carried out in the presence of fluconazole has also been associated with overall advantages in fitness [125]. Yet, azole-induced aneuploidies were lost during cultivation in a stress-free environment and were thus considered providers of raw genetic material in the process towards the acquisition of resistance. This ease for chromosomal changes also demonstrates and emphasizes the genomic plasticity of *Candida*.

Genome rearrangements have also been suggested to play a role in the adaptation of *C. glabrata* to stressful conditions [128]. Early studies from 1997 already reported whole chromosome duplications bearing the *ERG11* gene in azole-resistant strains [129]. Other investigations led to similar claims based on differences among karyotypes of serial clinical isolates of the species exhibiting an increased resistance to antifungals [130]. Yet, chromosomal aberrations were also observed in the same world-wide used *C. glabrata* reference strain obtained from various laboratories and cultivated under non-stressful conditions [131]. Similarly, in a recent whole genome sequencing analysis, aneuploidies containing genes involved in drug resistance were not associated with increased resistance profiles [63], again excluding a direct effect of chromosomal changes on antifungal drug resistance.

Thus, aneuploidies are acknowledged to play a role in mediating drug resistance in *C. albicans*, but the impact of this phenomenon in other species is not clear. On the other hand, alternative genomic changes involving copy-number variation (CNV), including short segmental CNV [64] and loss of heterozygosity (LOH) (in heterozygous species such as *C. albicans*), are also proposed to drive fast adaptation [81,132]. Furthermore, the appearance of ‘hypermutator phenotypes’ resulting from mutations in DNA repair genes has been proposed to precede the appearance of resistance in bacteria [133]. Similarly, hypermutator phenotypes resulting from mutations in the DNA mismatch repair gene *MSH2*, have been suggested to enable fast adaptation to drugs in *Cryptococcus neoformans* and *C. glabrata* [134,135]. However, at least for *C. glabrata*, other studies have cast doubts on the hypothesis that variations in *MSH2* generally precede the appearance of other resistance-conferring mutations. For instance, some of these mutations were found to be ancient polymorphisms within different *C. glabrata* clades, and to be equally widespread among non-resistant isolates [63]. Consistent with this, *MSH2* non-synonymous polymorphisms can be locally common, irrespective of the susceptibility of the isolates, as found for 69% (57/83) of susceptible clinical isolates in India [136].

Recent research has drawn attention to the existence of phenotypic variation within a genetically homogeneous population. This is particularly important for *C. glabrata* and its ability to undergo exposure to azoles. In this case, the so-called heteroresistant phenotype refers to the observation of the coexistence of various levels of resistance to antifungal drugs within a clonal cell population [137]. This trait may be a reason for the high natural tolerance of the species towards the drug, and it can actually be a mechanism that buys time until the appearance of mutations that confer a stable and constitutive resistance. The mechanism of heteroresistance is still poorly understood, as is its potential relationship with the evolutionary paths leading to antifungal resistance in *C. glabrata*. To complicate things further, heteroresistance might cause false outcomes in susceptibility tests, which may result in the misidentification of potentially resistant isolates as susceptible and even in fatal treatment failure [137]. Along with heteroresistance, we would like to mention the concepts of tolerance and persistence. Tolerance has been described as the ability of an organism to grow at concentrations higher than the MIC, in contrast to antifungal resistance that reflects the increase in MIC independent of the microorganisms’ capacity to survive at drug concentrations higher than this value [138,139]. Furthermore, tolerance is reversible and results from epigenetic mechanisms, while resistance is an inheritable property determined by genes and their mutations [139]. It has been observed that strains exhibiting tolerance are more prone to cause clinically persistent infections than strains having the same MIC but not being tolerant [140]. It has been also suggested that drug tolerance is an evolvable phenotype, which is distinct from and does not correlate with antifungal drug resistance [140]. Finally, persistence occurs when microorganisms are not only able to withstand the antifungal therapy, but can also cause a relapse, even after a successful one [140].

## 7. Whole Genome Sequencing of Serial Isolates to Track the Emergence of Resistance

The evolutionary paths leading to the appearance of resistance have been extensively studied in bacteria by means of whole genome sequencing of serial clinical isolates and in vitro evolution studies. Fortunately, nowadays, those approaches are also being increasingly introduced in the research of fungal pathogens. Sequencing the entire genome of a microorganism has never been so easy. Next-generation sequencing and comparative genomics not only allow us to record the footprints of genetic evolution of new species, but also help us in tracking the genomic changes that follow the emergence of a phenotype of interest. One of the initial studies on the genetic bases of yeast adaptation to antifungal drugs in a human host by means of genome sequencing was performed by Ford et al. [141]. In this study, sequencing was used to assess changes in the frequency of variants in *C. albicans* isolates sampled consecutively from the same patient and shown to acquire resistance by the end of the treatment. The study indicated persistent and recurrent LOH and SNPs in 166 genes as the main modifications associated with decreased fluconazole susceptibilities. More specifically, LOH was found on chromosome 3, in regions comprising *CDR1* and *CDR2* (efflux pumps coding genes) and *MRR1* (encoding the regulator of the *MDR1* major facilitator superfamily efflux pump) [142]; and on chromosome 5 with genes encoding the drug target *ERG11*, and *TAC1* (transcription factor that positively regulates the expression of *CDR1* and *CDR2*) [81]. Other mutations were found in cell adhesion genes (e.g., *ALS3,5* and *7* and *HYR3*), as well as genes involved in filamentous growth (e.g., *FGR14*, *FGR28*, and *EFH*) and biofilm formation (e.g., *BCR1* and *YAK1*), indicating that resistance was co-evolving with virulence. On the other hand, although aneuploidies were present and may be important adaptive intermediates (see above), they did not seem to correlate with the resistant phenotype. The authors maintain the suggestion that these variations ease the survival until more stable and/or less costly mutations arise. Additionally, it is also possible that serial isolates from the same patient can result in resistance caused by different trajectories [127]. Nine serial clinical *C. albicans* isolates obtained from a patient that underwent antifungal treatment were observed to acquire resistance by multiple and competing mechanisms [127]. This emphasizes the urge to understand the dynamics of emergence of the resistance, including the evolutionary trajectories, the rates at which different mutations arise, and the potential relationships between processes mediating the adaptive mechanisms.

Genome sequencing of serial clinical isolates has also been applied to *C. glabrata*. A recent study sequenced and compared the genomes of two *C. glabrata* clinical isolates obtained from the same patient separated by 50 days of azole treatment [143]. The identified genetic differences comprised 17 non-synonymous SNPs, including one gain of function substitution in the *PDR1* gene (L280F) and small-sized indels mainly affecting adhesin-like genes. Despite all the effort, which included the use of advanced PacBio long-read sequencing, the only significant mutation that was found was among those already known to confer azole resistance [144]. The rest of the observed genetic alterations were attributed to fitness or accidental mutations. Acquired resistance of *C. glabrata* to echinocandins was also analyzed by whole genome sequencing of serially isolated strains obtained from a patient subjected to caspofungin treatment [145]. This study identified non-synonymous mutations in the drug target gene *FKS2* and in other eight genes (the orthologs of *S. cerevisiae MOH1, GOH1, CDC6, TCB1/2, DOT6, MRPL11, SUI2*, and *CDC55*). Yet, the functions of the orthologs in *S. cerevisae* of these eight genes suggested that they were not directly related to the resistance phenotype, but rather that they might be connected to the adaptation of *C. glabrata* to the host or, alternatively, they might compensate for the effect of *FKS2* mutations. Additionally, changes in the *FKS2* gene were associated with the highest increase in echinocandin resistance and a considerable cost in fitness. Finally, another study in *C. glabrata* used a whole genome sequencing approach, but only searched for mutations in genes suggested to play a role in resistance [146]. More specifically *FKS1* and *FKS2* in echinocandin resistance; *FCY1*, *FCY2*, *FUR1*, *FPS1*, and *FPS2* in luorocytosine resistance; and *ERG9*, *ERG11*, *CDR1*, *PDR1*, *FLR1*, and *SNQ2* in azole resistance. Interestingly, the results uncovered specific mutations in *FKS1* (S629P) and *FKS2* (S663P) present only in the echinocandin-resistant strains. In contrast, mutations present in marker genes for azole resistance, *PDR1* and *CDR1*, were present in both azole-susceptible and resistant isolates, which again underscores the need for further investigations.

## 8. In Vitro Evolution Studies

Although in vivo studies performed on patient samples are clinically more relevant than in vitro ones, they come with disadvantages. In in vivo studies, the results are not easily replicable, the population size parameters are not controlled, and usually only the mutational composition of the final isolate is assessed. These limitations make the use of alternative in vitro approaches a promising tool to unravel the evolutionary paths leading to the emergence of resistance. This so-called ‘experimental evolution’ approach enables the control of conditions and exact measurement of relevant parameters. Moreover, samples can be stored at intermediate time points and the experiment can be re-started with alternative conditions from any point, thus allowing researchers to ‘retape’ evolution. Furthermore, the order of occurrence of adaptive mutations—i.e., the evolutionary trajectory—can be tracked. Several studies have shown a high consistency between results obtained in vivo and in vitro in yeasts like *C. albicans* and *S. cerevisiae* [147,148]. In vitro evolution experiments coupled with whole genome sequencing have been extensively used to understand the emergence of antibiotic resistance in bacteria [149], but their use in the field of antifungal drug resistance is still in its infancy.

**Figure 3:**
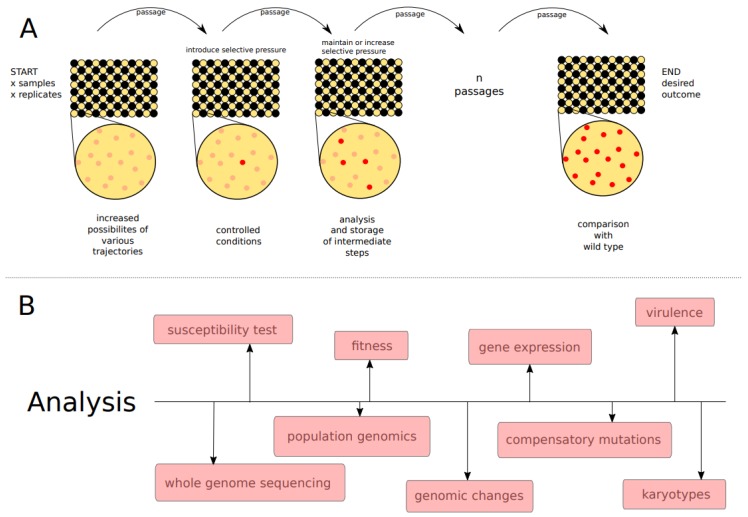
Schematic representation of an in vitro evolution experiment (**A**) and possible follow-up analysis (**B**). **A**–96-well plate can be inoculated in a checkerboard manner with up to 48 samples (sample—yellow well, blank—black well), allowing many possible combinations of strains and replicates. Initially, all cells within each population are expected to be genetically identical (enlarged well—pink circles). Next, the samples are introduced to a selective condition (for example antifungal drug). The amount of the sample (or number of growth cycles), the interval of the passages, and the amount of selective pressure between the passages can be set up and controlled as preferred. Ideally, each transfer favors a selection of mutants with a desired phenotype (red dots in the enlarged well). Storage and/or analysis of the samples can be performed as preferred, e.g., after each passage. The experiment is finished after a certain amount of time or when the desired phenotype is present in the evolving population. (**B**)–Further analysis subsequent to the in vitro evolution experiment may involve analysis of the genotype (top) or phenotype (bottom). These analyses can include, among others: drug susceptibility; fitness measurement (ability to replicate and survive in a given environment); assessment of levels of gene expression; virulence test (ability to infect or damage a host); whole genome sequencing; population genomics (large-scale comparison of DNA sequences of populations); identification of specific genomic changes (see Figure 2), with the possibility of determining compensatory mutations; karyotypes (changes in chromosome numbers or large genomic re-arrangements).

There are two main approaches for in vitro evolution experiments: batch serial transfer and continuous culture. In the first one, the sample is grown on selective solid or liquid media, and a fraction of it is repeatedly and serially transferred to a fresh medium. Then, the culture passes by different growth phases, which implies that the amount of nutrients in the medium diminishes with time. On the other hand, in a continuous culture system, the physiological state of the cells, the growth conditions, and the environment, including nutrient concentrations, are kept constant. Both methods have been successfully used to study the emergence of drug resistance in *Candida* yeasts [150,151]. The important advantages of a serial dilution system over the continuous culture are related to lower costs, the use of generally available laboratory equipment, and, most importantly, the feasibility of conducting experiments involving a high number of replicates in parallel, enabling a comprehensive analysis of a variety of changes, mechanisms, and evolutionary trajectories of adaptation. For example, Cowen et al. [150] serially propagated six experimental populations of *C. albicans* derived from a single colony for 330 generations in medium supplemented with fluconazole at a concentration doubling their MIC. This in vitro evolution experiment resulted in the selection of azole-resistant isolates evolved by different mechanisms and exhibiting distinct levels of resistance and different expression patterns for azole-associated genes (*CDR1*, *CDR2*, *ERG11*, and *MDR1*). Another study compared the evolution of experimental *C. albicans* populations evolved under the presence of fluconazole with those without this stress [151]. Similar to the previous study, multiple resistant mutants appeared rapidly in independent lineages. Moreover, they found that most adaptive mutants with increased fitness under drug exposure did not show significant fitness defects in the absence of the drug. These studies show the great potential of in vitro evolution studies for uncovering evolutionary paths leading to the emergence of resistance. Yet, such studies are scarce and limited to a few species and drugs, which suggests that many alternative adaptation pathways remain unknown. Hence, we believe that the use of in vitro evolution approaches, coupled with whole genome sequencing, should be extended in future studies. An example of an in vitro experimental evolution and a follow-up analysis is presented in Figure 3.

## 9. Conclusions 

Fungi pose a growing clinical threat, and we have limited drugs to combat them. The problem of resistance to antifungal drugs is highly prevalent and has increased over the last years. Currently, 20–30% of candidemia cases involve species with intrinsic resistance to either fluconazole or echinocandins [42]. This is a significant change as *C. albicans*, naturally susceptible to all drugs, used to account for 85% of the cases before the advent of antimycotics [42]. The main driver of this change involves the use, and overuse, of antifungal drugs in the clinics. Resistance can be based on diverse mechanisms, which can vary from species to species. *C. glabrata* is a good illustrating example of how a well-understood mechanism in one species does not necessarily apply to other species. Emerging and dangerous species, like multidrug-resistant *C. auris*, pose a constant threat and we are likely to witness the rise of new such multidrug-resistant pathogenic yeasts in the near future.

Next to the complexity of varying natural susceptibilities across species, we need to consider the process of secondary acquisition of resistance in otherwise susceptible yeasts. Such cases are being increasingly reported and have brought around an urgent need to develop more efficient ways to assess and monitor the microorganisms’ response to a drug, also during the course of the infection. More studies on the underlying processes of resistance and evolutionary pathways that result in drug adaptation are needed, as well-understood molecular mechanisms do not always completely account for the high levels of resistance observed in many clinical isolates. Fortunately, technical developments such as next-generation sequencing are allowing us to interrogate mutational processes at unprecedented levels of scale and resolution. Promising discoveries are being disclosed during the analysis of serially isolated clinical strains with acquired resistance and light is being shed on the complex landscape of mutations and genomic re-arrangements that lead to the emergence of the phenotype. Along with comprehensive sequencing and the comparison of clinical samples, several laboratories are approaching the issue by the use of controlled, experimental evolution experiments. Ongoing results are showing that rather than a single, established path, there is an array of possible trajectories by which a microorganism can adapt to drugs. Understanding the molecular and evolutionary mechanisms responsible for the development of drug resistance in common and emerging yeast pathogens will undoubtedly contribute to the development of novel target-specific drugs or resistance-blocking supplements. In addition, research on the genetic bases of resistance also has the potential to ultimately lead to novel diagnostic tools that would allow detecting particular resistant profiles from genetic hallmarks.

## Figures and Tables

**Table 1:** Modes of action of common antifungal drugs. Columns indicate, in this order: major classes of antifungal drug; drugs in clinical use; modes of action.

Antifungal Drug Class	Drug	Mode of Action
Azoles	Fluconazole	Inhibitor of lanosterol 14α—demethylase
	Voriconazole	
	Posaconazole	
	Itraconazole	
	Ketoconazole	
	Clotrimazole	
	Econazole	
	Miconazole	
Echinocandins	Caspofungin	Inhibitor of 1,3–β–glucan synthase
	Anidulafungin	
	Micafungin	
Polyenes	Amphotericin B	Binding to ergosterol
	Nystatin	
Pyrimidine analogue	flucytosine	Inhibitor of DNA/RNA/protein synthesis

**Table 2:** Intrinsic susceptibility patterns in *Candida* and *Saccharomyces cerevisiae.* Letters indicate susceptibility categories based on EUCAST (European Committee on Antimicrobial Susceptibility Testing) breakpoints: S—Susceptible, I—Intermediate, R—Resistant. In the absence of an established breakpoint, X indicates species with elevated minimum inhibitory concentrations (MICs) compared with *Candida albicans*. The four most common *Candida* are indicated in bold. (adapted from [42]).

	Fluconazole	Echinocandins	Amphotericin B
***Candida albicans***	S	S	S
*C. auris*	X	X	X
*C. cifferrii*	X		
*C. dubliniensis*	S	S	S
*C. duobushaemulonii*	X	X	X
*C. fermentati*		X	
***C. glabrata***	I	S	S
*C. guilliermondii*	X	X	
*C. haemulonii*	X	X	X
*C. humicola*	X		
*C. inconspicua*	X		
*C. krusei*	R	S	S
*C. lambica*	X		
*C. lipolytica*	X	X	
*C. lusitaniae*			X
*C. metapsilosis*		X	
*C. norvegensis*	X		
*C. orthopsilosis*		X	
*C. palmioleophila*	X		
***C. parapsilosis***	S	I	S
*C. pseudohaemulonii*	X	X	X
*C. rugosa*	X		
***C. tropicalis***	S	S	S
*C. valida*	X		
*S. cerevisiae*	X		

**Table 3:** Genetic bases of resistance towards common antifungal drugs. Columns indicate, in this order: drug class, mode of resistance, genes involved, species for which this resistance mode has been found (with four major pathogenic species in bold), and comments.

Antifungal Drug Class	Mode of Resistance	Gene	Species	Comments
Azoles	drug target overexpression → increased concentration of lanosterol 14α—demethylase	*ERG11*	***C. albicans*** ***C. parapsilosis*** ***C. tropicalis*** *C. krusei*	overexpression regulated by *UPC2*
	drug target alteration → decreased lanosterol 14α—demethylase binding affinity for the drug	*ERG11*	***C. albicans*** ***C. parapsilosis*** ***C. tropicalis*** *C. krusei* *C. auris*	
	aneuploidy	*ERG11, UPC2, TAC1*	***C. albicans***	
	loss of heterozygosity	*ERG11, TAC1, MRR1*	***C. albicans***	
	drug counteraction → inactivation of C5 sterol desaturase leading to alterations in the ergosterol synthetic pathway → reduction of ergosterol and accumulation of other sterols	*ERG3*	***C. albicans***	
	overexpression of drug transporter (efflux pumps)	*CDR1, CDR2, SNQ2, ABC1*	***C. albicans*** ***C. parapsilosis*** ***C. tropicalis*** *C. krusei* ***C. glabrata***	ATP binding cassette (ABC transporter), regulated by *TAC1, PDR1*
		*MDR1, TPO3*	***C. albicans*** ***C. parapsilosis*** ***C. tropicalis*** ***C. glabrata***	Major facilitator family (MFS transporter), regulated by *MRR1*
Echinocandins	drug target alteration → decreased glucan synthase processivity for the drug	*FKS1*	see Table 4	
		*FKS2*	*Merged*	
Polyenes	Frame shift mutation	*ERG2*	***C. albicans***	cross resistance to azoles
	point alteration → decreased ergosterol content in cells	*ERG2*	***C. glabrata***	cross resistance to azoles
		*ERG3*	***C. albicans***	cross resistance to azoles
		*ERG5*	***C. albicans***	cross resistance to azoles
		*ERG6*	***C. glabrata***	
		*ERG11*	***C. albicans***	cross resistance to azoles
Pyrimidine analog	point alteration → inactivation of cytosine permease affecting drug uptake	*FCY2*	*C. lusitaniae* ***C. glabrata***	
	point alteration → inactivation of cytosine deaminase leading to alterations in the metabolism of 5-fluorocytosine	*FCY1*	***C. glabrata***	
	point alteration → inactivation of uracyl phosphoribosyl transferase leading to alterations in the metabolism of 5-fluorocytosine	*FUR1*	***C. albicans***	
	Deletion → reduced accumulation of the drug	*FPS, FPS2*	***C. glabrata***	

**Table 4:** Point mutations in hotspots of *FKS1* and *FKS2* genes connected with resistance towards echinocandins in *Candida* and *Saccharomyces cerevisiae*. Columns indicate, in this order: organism, with the four major pathogens indicated in bold; if applicable, intrinsically lower susceptibility (X); and for *FKS1* and *FKS2* hotspots, respectively, the starting amino acid position and the sequences of interest. One letter codes are used for the amino acid sequence, with colors pointing to sites that are mutated. Mutations are marked: red as strong, orange as weak, green as silently acquired or naturally occurring, blue as naturally intrinsic proven or possibly related to the intrinsic lower susceptibility, and violet as naturally occurring of unknown impact. Further, * indicates the codon involving a mutation or deletion and ** codon involving a mutation or a stop codon (adapted from: [34]).

Organism		FKS1				FKS2			
		Start	HOT SPOT 1	Start	HOT SPOT 2	Start	HOT SPOT 1	Start	HOT SPOT 2
***Camdida albicans***		641	FLTLSLRDP	1357	DWIRRYTL				
*C. dubliniensis*		641	FLTLSLRDP	1357	DWIRRYTL				
***C. glabrata***		625	FLILSLRDP	inaccurate	DWVRRYTL	659	F*LILSLRDP	1374	DWIR**RYTL
*C. kefyr*		inaccurate	F*LTLSLRDP	inaccurate	DWVRRYTL				
*C. krusei*		655	FLILSIRDP	1364	DWIRRYTL				
*C. lusitaniae*		inaccurate	FLTLSLRDP	inaccurate	DWIRRYTL				
***C. tropicalis***		inaccurate	FLTLSLRDP	inaccurate	DWIRRYTL				
***C. parapsilosis***	X	652	FLTLSLRDA	1369	DWIRRYTL				
*C. metapsilosis*	X	inaccurate	FLTLSLRDA	inaccurate	DWIRRYTL				
*C. orthopsilosis*	X	inaccurate	FLTLSLRDA	inaccurate	DWVRRYTL				
*C. guiliermondii*	X	632	FMALSLRDP	1347	DWIRRYTL				
*C. lipolytica*	X	662	FLILSLRDP	1387	DWIRRCVL				
*S. cerevisae*		639	FLVLSLRDP	1353	DWVRRYTL	658	FLILSLRDP	1372	DWVRRYTL
